# Protocol for a randomized controlled trial comparing a very low-carbohydrate diet or moderate-carbohydrate plate-method diet for type 2 diabetes: the LEGEND (Lifestyle Education about Nutrition for Diabetes) trial

**DOI:** 10.1186/s13063-023-07512-9

**Published:** 2023-07-20

**Authors:** Laura R. Saslow, Adriana Eslamian, Patricia Moran, Wendy Hartogensis, Ashley E. Mason, Sarah Kim, Douglas C. Bauer, Dina Hafez Griauzde, Veronica Goldman, Vivian Liu, Pam Stephens, Kate Raymond, George Yeung, Cindy Leung, Frederick M. Hecht

**Affiliations:** 1 University of Michigan, Ann Arbor, MI USA; 2 University of California, San Francisco, CA USA; 3 Harvard University, Cambridge, MA USA

**Keywords:** Type 2 diabetes, Glycemic control, Lifestyle intervention, Randomized controlled trial

## Abstract

**Background:**

Optimal carbohydrate intake is an important and controversial area in the nutritional management of type 2 diabetes. Some evidence indicates that reducing overall carbohydrate intake with a low- or very low-carbohydrate eating plan can improve glycemic control compared to following eating plans that involve greater carbohydrate intake. However, critical knowledge gaps currently prevent clear recommendations about carbohydrate intake levels.

**Methods:**

The LEGEND (Lifestyle Education about Nutrition for Diabetes) Trial aims to compare a very low-carbohydrate diet to a moderate-carbohydrate plate-method diet for glycemic control in adults with type 2 diabetes. This two-site trial plans to recruit 180 adults with type 2 diabetes. We will randomize participants to either a 20-session group-based diet and lifestyle intervention that teaches either a very low-carbohydrate diet or a moderate-carbohydrate plate-method diet. We will assess participants at study entry and 4 and 12 months later. The primary outcome is HbA1c, and secondary outcomes include inflammation (high sensitivity C-reactive protein), body weight, changes in diabetes medications, lipids (small particle LDL, HDL, triglycerides), skeletal metabolism (bone mineral density from dual-energy x-ray absorptiometry and bone turnover markers serum procollagen type I N propeptide and serum C-terminal telopeptide of type I collagen), and body composition (percent body fat, percent lean body mass).

**Discussion:**

The LEGEND trial is a randomized controlled trial to assess optimal carbohydrate intake in type 2 diabetes by evaluating the effects of a very low-carbohydrate diet vs. a moderate-carbohydrate plate-method diet over a year-long period. The research addresses important gaps in the evidence base for the nutritional management of type 2 diabetes by providing data on potential benefits and adverse effects of different levels of carbohydrate intake.

**Trial registration:**

ClinicalTrials.gov NCT05237128. Registered on February 11, 2022

## Introduction

### Background and rationale {6a}

Type 2 diabetes is the most expensive chronic disease in the USA, with annual costs currently estimated to be more than $325 billion [1]. Nutritional management of type 2 diabetes holds potential to improve glycemic control and clinical outcomes but there is a lack of high-quality research to inform practice guidelines on how to optimize nutritional management of type 2 diabetes [2]. In 2019, the American Diabetes Association’s Nutrition Review Committee concluded that “Despite widespread interest in evidence-based diabetes nutrition therapy interventions, large, well-conducted nutrition trials continue to lag far behind other areas of diabetes research” [3].

Optimal carbohydrate intake is an important and controversial area in the nutritional management of type 2 diabetes. Some evidence indicates that reducing overall carbohydrate intake with a low- or very low-carbohydrate eating plan can improve glycemic control compared to eating plans with greater carbohydrate intake [2]. However, three critical knowledge gaps currently prevent clear recommendations in guidelines for carbohydrate intake. First, there is a gap in knowledge about the durability of gains in glycemic control with low-carbohydrate diets [4, 5]. It is currently unclear how much the loss of improvements is due to reduced adherence that may be readily addressed with appropriate behavioral strategies. Second, there are concerns about possible long-term adverse effects on lipids [6] and bone mineral density [7]. Third, although current recommendations from the American Diabetes Association’s Nutrition Review Committee suggest individualizing recommendations about carbohydrate intake, we have almost no knowledge to guide such individualization.

### Objectives {7}

To address these critical gaps in the evidence base for optimal carbohydrate intake recommendations for type 2 diabetes, we are conducting a two-site, parallel-group, randomized (1:1) trial with a 12-month follow-up in 180 adults with type 2 diabetes, comparing a very low-carbohydrate diet to a moderate-carbohydrate plate-method diet. The American Diabetes Association recommends both dietary approaches. Both dietary groups will be taught previously tested behavioral strategies to maintain adherence to nutritional treatment, including relapse-prevention planning, self-monitoring, and social support [8]. This trial aims to compare the long-term effects of a very low-carbohydrate to a moderate-carbohydrate diet on clinical outcomes including glycated hemoglobin (HbA1c, primary outcome), weight, body composition, inflammation (high sensitivity C-reactive protein), ability to reduce the use of diabetes medications and medication-related costs, and diabetes-related distress over 12 months. We hypothesize that the very low-carbohydrate diet group (as compared to the moderate-carbohydrate diet group) will have improvements in HbA1c and other outcomes at 12 months. We will compare the adverse effects associated with a very low-carbohydrate diet to those associated with a moderate-carbohydrate diet. We hypothesize that the two dietary approaches will have similar effects on lipids and bone metabolism and will be associated with similar side effects (such as constipation and headaches). We will also explore changes in vegetable and fruit consumption. Finally, we will assess factors that identify individuals who will particularly benefit from one or the other dietary approaches. We will explore whether baseline characteristics modify the benefits of diet group assignment on HbA1c by assessing whether there appear to be differences in the magnitude of HbA1c changes between diet groups across three pre-defined sub-groups (levels of insulin resistance, levels of obesity, and women versus men).

### Trial design {8}

This trial is a two-site, parallel-group, randomized controlled trial comparing two different dietary approaches for type 2 diabetes: a very low-carbohydrate diet vs. a moderate-carbohydrate plate-method diet. We will randomize a total of 180 adults with type 2 diabetes using an allocation ratio of 1:1. This is a superiority trial. Assessments occur at baseline and 4 and 12 months after baseline.

## Methods: participants, interventions, and outcomes

### Study setting {9}

The trial includes two clinical sites at university medical center health systems. One site is at the University of Michigan, Ann Arbor, MI (L. Saslow, PI), and the other site is at the University of California, San Francisco (F. Hecht, PI). We will recruit participants from the University of Michigan and University of California, San Francisco, health systems using outreach email and letters to potentially eligible participants identified using electronic health records at these sites. We will also advertise on social media to do outreach to populations living in the Ann Arbor, Michigan, and San Francisco, California, areas. We aim to enroll a generally nationally representative sample of persons with type 2 diabetes.

### Eligibility criteria {10}

Key inclusion criteria include the following: (1) diagnosis of type 2 diabetes, with current HbA1c ≥ 6.5% and <12% (measured using a blood sample collected at screening); (2) ability to speak English; (3) age 21 or older; (4) ability to engage in light physical activity; and (5) willingness to be randomized to either type of diet. Exclusion criteria include the following: (1) unable to provide informed consent; (2) substance abuse, mental health, cognitive, or medical conditions that would, in the opinion of the investigators, make it difficult for the individual to take part in the intervention, may alter key outcomes, or may require important diet modifications and includes conditions such as hypercalcemia or thyroid dysfunction; (3) pregnant or planning to become pregnant in the next 12 months or currently breastfeeding (self-reported; pregnancy and breastfeeding require modifications to the intervention’s dietary approaches; participants of child-bearing potential with a positive urine pregnancy test prior to dual x-ray absorptiometry (DXA) scan will be ineligible); (4) current use of weight loss medications (self-reported) as this is likely to alter weight outcomes and may alter other measures; (5) history of weight loss (bariatric) surgery or plans for bariatric surgery in the next year (self-reported); (6) currently enrolled in a weight loss program or have unalterable plans to enroll in a diet or weight loss programs in the next year (self-reported in screening survey); (7) vegan or vegetarian (self-reported in screening survey) as this requires additional modification of the group-based nutrition education curriculum; (8) unwilling or unable to participate in study measurements and group classes; (9) current use of systemic steroids or immunomodulators (self-reported in screening survey; oral or IV systemic steroids are excluded but local injected steroids may be permitted if frequency and timing of injections allows for at least of 2 weeks of spacing with blood tests; (10) above weight limit for DXA scanners (400 pounds); (11) history of multiple or recent (within the last 4 years) kidney stones; (12) currently following a very low-carbohydrate diet; and (13) unwilling to stop an SGLT2 inhibitor medication if the participant were to be randomized to the very low-carbohydrate diet.

### Who will take informed consent? {26a}

A member of the study team with appropriate ethical training and institutional review board approval will describe the consent forms in a detailed way and clarify any questions participants may have. The study team will describe that the participation in the study is voluntary.

### Additional consent provisions for collection and use of participant data and biological specimens {26b}

We will collect blood samples to assess outcomes such as HbA1c, high-sensitivity C-reactive protein, lipids, bone turnover markers, and insulin resistance. However, there will be no storage of biological samples.

## Interventions

### Explanation for the choice of comparators {6b}

We will compare two eating patterns diabetes; one is a very low-carbohydrate diet and the other is a moderate-carbohydrate plate-method diet. Both nutrition approaches are recommended by the American Diabetes Association, but there is limited rigorous data comparing the effects of these two nutrition plans, which primarily vary in carbohydrate levels, over a 12-month period.

All participants will receive nutritional instruction and training in basic behavioral strategies through live internet video-based sessions, meeting weekly (except if this falls on a holiday) to cover the eleven 1-h core sessions in diet-specific groups. We aim to include approximately 30 people in each cohort in order to have about 15 in each class. After the first 11 core sessions, participants will meet monthly for the remaining nine months of the study participation period (nine 1-h maintenance sessions). Sessions will be led by teachers experienced in each dietary approach, one teacher per group. A member of the study team will also be available during class sessions to answer logistical questions and provide technological support. Participants will receive printed study materials in the mail at the start of the intervention, as well as electronic copies of class materials prior to each class.

During the core phase of the intervention (first 11 weekly sessions), all participants will be sent brief (2–5 min) weekly check-in surveys. These surveys will ask participants whether they plan on attending the session the following week, if they have experienced any health symptoms, and their adherence to the dietary approach. Participants on glucose-lowering medications will be asked if they have had very high or very low blood glucose results and/or symptoms of hypoglycemia; if yes, they will be asked to provide their recent results and current diabetes medications so that the study team can follow-up with them to adjust medications or troubleshoot if needed. During the maintenance phase (remaining nine months of study participation), participants will complete a check-in survey monthly, prior to each session.

## Intervention description {11a}

### Very low-carbohydrate diet

This dietary approach will encourage a very low-carbohydrate (ketogenic) eating pattern that aims to reduce carbohydrate intake to 20–35 non-fiber grams of carbohydrates a day. This dietary approach aims to reduce carbohydrate intake to a point that induces a low level of ketone production. Nutritional ketosis may serve as a marker indicating that insulin levels are reduced enough to allow the body to begin using fat as a key source of energy, reducing inhibition of lipolysis by insulin. When this occurs, some fats are turned into ketones, which serve as a readily used fuel. Participants randomized to this diet group will be mailed urine ketone strips. They will be encouraged to use ketone urine test strips at the beginning of their time following this dietary approach to help them gauge whether they are achieving nutritional ketosis. Most calories are derived from meat, fish, full-fat dairy, eggs, fats, nuts, seeds, oils, leafy, or other low-carbohydrate vegetables (such as spinach, lettuce, asparagus, eggplant, cabbage, kale, Brussels sprouts, green peppers, and green beans), and low-carbohydrate fruits (such as raspberries and blackberries). Participants will be advised to eliminate most starches and sweets such as potatoes, rice, pasta, bread, donuts, and sugar-sweetened beverages. Participants will be advised to eat protein with each meal and derive their remaining calories from fat.

### Plate-method diet

The “*Create Your Plate*” meal planning approach has been developed by the American Diabetes Association and represents a diet currently recommended for type 2 diabetes in many settings [9]. The plate is commonly used as a tool for teaching people how to reduce starches while balancing meals with protein and vegetables. Patients are encouraged to keep their starches to ¼ of their plate, have protein foods fill another ¼ of the plate, and fill the remaining ½ with non-starchy vegetables. This instructional approach can be useful in working with people with low literacy or with various cultural cuisines. The focus of the plate is whole foods, rather than processed foods. This method also emphasizes getting most nutrition through meals, rather than consuming calories from liquids. Eating food, particularly with fiber, rather than consuming calories via liquids, may be a strategy to reduce postprandial blood glucose, and fiber in food may help with satiety between meals. The overall carbohydrate intake in this meal planning approach (about 45–48% of calories from carbohydrates in many typical meals) [10] is similar to that typically consumed by people with type 2 diabetes in the USA; one large study found that the average carbohydrate intake by people with type 2 diabetes was 44% of calories (about 190–280 g/day) [11].

### Physical activity and sleep goals

Both diet groups receive the same advice for physical activity and sleep. The physical activity goal for this program is a total of 150 min per week of moderate-intensity exercise, similar in intensity to brisk walking. This is the same target as was used in the Diabetes Prevention Program [8]. We also recommend that participants acquire adequate sleep, describing that most adults need 7–9 h of sleep per night [12].

### Behavioral goals

Both diet groups receive the same core cognitive and behavioral therapy intervention-based components similar to those used in the Diabetes Prevention Program [8]. Core components that have demonstrated effectiveness in changing health behavior in ways that achieve benefits in clinical outcomes include self-monitoring of diet, increasing problem-solving skills, and goal setting [13]. For example, teachers guide participants through a goal-setting exercise and introduce self-monitoring. Self-monitoring strategies are tailored to each intervention arm. For the very low carbohydrate group, self-monitoring focuses on using urine-based test strips as feedback to achieve target carbohydrate restriction levels. For the plate-method group, the focus is on adherence to the plate-method approach. All topics by session number are in Table 1.

**Table 1:** Class schedule for common elements in both intervention arms

**Class/session**	**Topics**
**Core sessions**	
#1	Study description, rationale of assigned diet, class expectations, personal goals for joining the study, changing snacks and breakfasts, tracking diet, side effect guide
#2	Changing lunches, reading nutrition labels, calculating net carbs and calories, menu makeover
#3	Changing dinners, grocery store walkthrough, sugar and artificial sweeteners, meal prepping, food substitutions, tracking urinary ketones (for keto group)
#4	How to plan and eat when dining out or traveling
#5	Recognizing and changing food cues and habits, binge eating and trigger foods, alcohol
#6	Physical activity and sleep
#7	Weight loss plateaus, how to find support and inspiration when dealing with challenges
#8	Guide for problem solving
#9	Saturated, unsaturated, and trans fats
#10	Slip-ups and how to deal with them
#11	Progress overview and ways to stay motivated
**Maintenance sessions**	
#1	Self-assessment and adjustments, facing external realities
#2	Potential causes of stress and how to make a plan for coping with different stresses
#3	Revisiting recipes and cooking, creative and easy recipe ideas
#4	Handling holidays, vacations, and special events; responding to questions and social pressure
#5	The relapse chain and strategies for preventing relapse, inspiration for recommitting to diet, grocery shopping list
#6	Dealing with social cues and social events, finding support from others
#7	Overcoming negative thoughts by learning how to talk back to them
#8	Stress and time management, taking charge of stress response, saving time with physical activity, making time for laughter and relaxation
#9	Ways to counter self-defeating thoughts, working on not giving into excuses and rationalizations, personal goals moving forward, sharing progress and success stories

### Criteria for discontinuing or modifying allocated interventions {11b}

Serious adverse events (other than short-term and correctable hypoglycemia) as a result of the intervention are not expected, but should they occur, participants will stop the intervention and be included in the “intention to treat” analysis for the primary endpoint. Criteria for discontinuing or modifying the intervention include changes in the therapeutic plan of participants, such as the need to initiate a protein-restricted diet. Participants will also be informed that they may refuse to answer any questions asked as part of outcome measures by the study team and that they may withdraw their consent to participate in the study at any time.

### Strategies to improve adherence to interventions {11c}

Both dietary programs include strategies to improve participant adherence such as goal setting and problem-solving. Intervention fidelity measures include coded, audiotaped sessions and surveys about each group’s teachers. Data from these assessments will be discussed as needed with the study team and diet group teachers. Additional training for the teachers may occur if deemed necessary by the study investigators.

### Relevant concomitant care permitted or prohibited during the trial {11d}

Participants will be asked to adhere to their assigned diet plan but are not restricted from engaging in any other treatments.

### Provisions for post-trial care {30}

We will not be providing post-trial care. We do not anticipate harm and therefore no compensation for harm due to trial participation.

## Outcomes {12}

### Primary outcome

#### HbA1c

HbA1c is the most widely accepted measure of overall glycemic control in clinical care of type 2 diabetes and is a strong predictor of the risk of microvascular complications from diabetes [14]. We will measure HbA1c levels using standard immunoturbidimetric assay methods and quality control measures at a Clinical Laboratory Improvement Amendments (CLIA) certified lab (i.e., Labcorp). Change in HbA1c over 12 months is our primary outcome; change in HbA1c from baseline to month 4 is a secondary outcome.

### Secondary outcomes

#### High sensitivity C-reactive protein

Increased inflammation is a hallmark of metabolic syndrome and is hypothesized to be a factor in the pathogenesis of macrovascular disease in diabetes. Beta hydroxybutyrate, the most abundant ketone body during a ketogenic diet, inhibits assembly of the NLRP3 inflammasome, thus decreasing release of inflammatory mediators from macrophages [15, 16]. A ketogenic diet also reduces reactive oxygen species, which are linked to inflammatory diseases [17]. High sensitivity C-reactive protein (hsCRP) is an important acute phase protein (inflammation protein). It is associated with increased risk of cardiovascular disease [18]. This measure will be assessed using standardized methods in a CLIA certified laboratory (Labcorp).

#### Weight

Weight loss, even in modest amounts, predicts improved glycemic control in type 2 diabetes [19]. Participants will be weighed at their in-person DXA appointments at baseline and 12 months. At 0, 4, and 12 months, we will collect measurements from their home scale.

#### Diabetes medications

We will ask participants about their current diabetes-related medication regimen using questions we have employed in prior studies [20]. We will assess estimated costs of diabetes-related medications in each diet group, using published cost data and will compare intensity of treatment needed in each arm using a medication-effect score, which assesses overall intensity of a diabetes medication regimen by combining dosage and strength of medications [21].

#### Lipids

Given that the very low-carbohydrate diet involves increasing the proportion of calories derived from fat compared to a conventional diet for diabetes, there have been concerns about the effects of this diet on lipids, particularly LDL cholesterol. Studies of very low-carbohydrate diets in the general population have found modest increases in LDL. Understanding the effects of a ketogenic diet on major plasma lipid fractions (HDL, LDL, triglycerides) and LDL particle size is important in assessing the possible impact of the diet on macrovascular complications in type 2 diabetes. We will measure triglycerides and fractionated cholesterol using Labcorp’s nuclear magnetic resonance (NMR) LipoProfile [22]. This advanced lipid assay provides measures that are more tightly tied to elevated cardiovascular risk than conventional lipid assays [23].

#### Bone mineral density

There is some prior evidence that ketogenic diets may have adverse effects on bone mineral density, presumably mediated via changes in bone metabolism, but data on this effect in adults are limited. Bone mineral density assessed by DXA is the preferred method for measuring of bone loss and estimating fracture risk, but treatment or environmental effects may not reliably be detected by DXA for months to years.

#### Bone turnover markers

Changes in biochemical markers of bone turnover respond to changes in bone metabolism over weeks to months and have been extensively used to better understand the causes of bone loss [24]. Two markers are recommended for clinical practice and skeletal research: one for bone formation (serum procollagen type I N propetide, s-PINP) and one for bone resorption (serum C-terminal telopeptide of type I collagen, s-CTX) [24]. DXA scans will be performed following recommended best practices for determining bone mineral density [25]. Automated assays of s-PINP and s-CTX will be performed using standardized methods in a CLIA certified laboratory (Labcorp).

#### Body composition

Weight loss may involve loss of lean and/or fat body mass, which have differing relationships to health; less lean body mass has been tied to greater mortality [26]. We will measure body composition using DXA scans.

### Other assessments

#### Diabetes-related distress

In addition to typical stressors, individuals with type 2 diabetes experience added psychological stress due to their diabetes diagnosis and its management. The Diabetes Distress Scale [27] is a 17-item scale that measures distress related to having diabetes.

#### Health-related quality of life (HRQoL)

We will use the PROMIS 29 [28], a psychometrically sound, clinically relevant measure [29, 30]. The PROMIS 29 assesses seven domains: physical functioning, anxiety, depression, fatigue, sleep disturbance, social functioning, and pain.

#### Sweet food cravings and emotional eating

We will assess food craving and emotional eating with the following scales: the craving for sweet subscale of the Control of Eating Questionnaire [31] and the coping subscale of the Palatable Eating Motives Scale [32].

#### Diet-related symptoms

It is important to understand whether the study’s dietary approaches cause physical health symptoms. We will monitor for these symptoms throughout the trial. At baseline and 4 and 12 months, participants will complete a symptom checklist. Participants report how often over the past month (no days, one day, several days, more than half the days, and nearly every day) they had symptoms such as dizziness, shortness of breath, headaches, general aches and pains, heartburn or acid reflux, constipation, and diarrhea.

#### Dietary adherence and changes

A day or two before each of the 20 classes, we ask participants “How well have you been following your assigned diet plan this week? On a scale of zero to ten, with zero being not at all, five being somewhat, and ten being following the plan very well, where you would place yourself?” In addition, at months 4 and 12, participants answer the following question: “How well have you been following your assigned way of eating? On a scale of zero to ten with zero being not at all, five being somewhat, and ten being following the plan very well, where would you place yourself?” We will consider a participant adherent to their diet plan at month 12 if the final four questions about dietary adherence (the last 3 questions tied to class attendance as well as the month 12 question) average at least a 7. In addition, we will assess dietary changes with one unannounced 24-h dietary recall at baseline and 4 and 12 months, which allows us to measure the absolute and percent of calories of each macronutrient.

#### Fruit and vegetable consumption

Fruit and vegetable consumption, particularly vegetables, have been linked to improved health outcomes [33]. Specifically, we will examine fruit juice (which include citrus juice), fruit (which includes citrus, other fruits, fried fruits, and fruit-based savory snacks), starchy vegetables (which includes potatoes, fried potatoes, legumes, fried vegetables, vegetable-based savory snack, and other starchy vegetables), avocados, dark-green vegetables, and other vegetables (deep-yellow vegetables, tomatoes, and other vegetables). The University of Michigan Nutrition Obesity Research Center has a Nutrition Assessment Laboratory, whose staff is available to perform gold standard nutrition assessments using 24-h dietary recalls utilizing the USDA 5 pass method. Participants will complete one 24-h dietary recall at baseline and 4 and 12 months. All recalls are analyzed using the Nutrition Data System for Research, a well-researched and developed program specifically designed for the collection and analysis of such recalls. Using information from the unannounced 24-h dietary recalls, we will explore the extent to which fruit and vegetable intake changes in the two study arms.

### Diabetes remission

We will explore whether participants meet criteria for diabetes remission, here defined as a 12-month HbA1c level of <6.5% while taking no glucose-lowering medications for the 3 prior months [34]. We will also explore whether participants meet criteria for diabetes reversal, here defined as a 12-month HbA1c level of <6.5% while taking no glucose-lowering medications or just metformin at the 12-month time point [35].

### Moderators

#### Insulin resistance

During insulin resistance, higher insulin levels are required to keep glucose at normal levels, and levels of insulin resistance vary. Some previous research has found that insulin-resistant adults lose more weight when assigned to a lower versus higher carbohydrate diet, although results are mixed [36–40]. We will use fasting insulin and glucose to estimate insulin resistance by calculating Homeostatic Model Assessment-Insulin Resistance (HOMA-IR). HOMA-IR provides a widely used method of estimating insulin resistance from a single fasting blood draw that has been reported in over 500 studies and correlates well with results from a hyperinsulinemic euglycemic clamp [41].

#### Body mass index

We will assess participants’ height and weight at baseline, to assess baseline body mass index.

#### Biological sex

In previous research, sex has modified the effect of diet type on weight loss such that men lost more weight in a low-carbohydrate versus high-carbohydrate diet compared to women [42]. However, the impact of biological sex on glucose control has not been previously reported to our knowledge. We will assess sex at baseline.

#### Participant timeline {13}

##### Screening survey

Participants will be directed via recruitment materials to a screening survey online (Qualtrics), which will briefly describe the trial and ask for limited consent. All recruitment materials will have study staff contact information so that interested participants can reach out via phone or email to ask questions about the trial. If needed, study staff may follow-up with participants to clarify screening survey responses. For example, if a participant is near the weight limit, or if a participant skips answers to one or more questions, study staff will reach out to the participant to follow-up. Study staff may ask participants who have not had HbA1c tested in the past several months to get an HbA1c test from their primary care physician (PCP) before proceeding further with the screening steps.

##### Orientation video and survey

Participants who appear eligible based on the screening survey will receive a link to an orientation video. Participants will be asked to watch the video, which details study procedures (including the randomization process and time requirements). Participants will then complete a short survey that includes comprehension questions about what they learned in the video, as well as the pros and cons of participating for them, whether they continue to be interested in participating in the trial.

##### Phone screen, consent for further screening, and second screening survey

After completing the orientation video and survey, study staff will contact interested participants by phone to answer any questions participants may have and to confirm participants’ initial eligibility by clarifying answers to the screening survey. Study staff will briefly explain the remaining screening steps over the phone and email eligible and interested participants a Consent for Further Screening consent form via DocuSign (California) or SignNow (Michigan). After participants sign the Consent for Further Screening, they complete a second screening survey (Qualtrics) with questions about medical history as well as drug and alcohol use.

##### Screening lab tests

If participants remain eligible after completing the second screening survey, they will be asked to get blood drawn at a commercial medical laboratory (Labcorp) for eligibility blood tests. Participants will be instructed to fast for 12 h prior to this appointment. Eligibility tests include the following: HbA1c (to confirm it is in the type 2 diabetes range), thyroid-stimulating hormone (to check for normal thyroid function), and a comprehensive metabolic panel (to check liver and kidney function). Participants who are taking insulin or who answer positively to at least 2 out of 4 Latent Autoimmune Diabetes in Adults screening questions [43] will receive an insulin C-peptide test during this screening blood draw. To be eligible to continue, participants must have an HbA1c between 6.5 and 11.9%, thyroid-stimulating hormone in a normal range, and liver and kidney function in a normal range. Participants who are taking insulin and/or screened positive for Latent Autoimmune Diabetes in Adults and who have a C-peptide below 1.0 ng/mL are ineligible. If C-peptide is between 1.0 ng/L and 1.5 ng/mL, these participants will return to Labcorp for GAD65 testing. Positive GAD65 results confer ineligibility given likely Latent Autoimmune Diabetes in Adults. The study team will inform participants of their eligibility based on their lab results. Participants who are eligible based on labs will be invited to schedule a virtual visit to sign the full-study consent form.

##### Trial consent visit (virtual) and preparation for randomization

For participants still interested and eligible based on the above steps, study staff will set up a virtual study consent visit via video chat. In this interaction study staff will review class schedules, the randomization process, assessments, and expectations and answer any questions. Participants will sign the full study consent form via SignNow (University of Michigan) or DocuSign (University of California) and will receive a signed copy for download to their email. After signing the consent form, participants will schedule their in-person DXA scan appointment. The study team will use HIPAA-compliant virtual faxing services or direct messages in MiChart (for Michigan Medicine providers) to send PCPs notice of their patient’s likely enrollment in the trial with a brief explanation of possible medication adjustments and the two study diet groups.

After completing the trial consent visit, participants will complete the following baseline assessments before being randomization to one of two diet groups:

- 24-h dietary recall: Participants will be contacted by phone unannounced for one 24-h dietary recall from trained, masked staff, using current Nutrition Data System for Research software.
- Baseline blood draw: Participants will go to a Labcorp blood draw location for a fasting blood draw that includes HbA1c, insulin, plasma glucose, high-sensitivity C-reactive protein (hsCRP), nuclear magnetic resonance LipoProfile, blood ketones, serum procollagen type I N propetide (s-PINP), and serum C-terminal telopeptide of type I collagen (s-CTX).
- DXA of the hip and spine, body composition: Participants of child-bearing potential will complete a urine pregnancy test prior to receiving their scan. Only participants with negative pregnancy tests will proceed with enrollment.
- Baseline self-report survey: Approximately 2 weeks before classes begin, participants will complete a baseline self-report survey online via Qualtrics, which takes approximately 30 min to complete. This includes questions about mood, health history, medications, diabetes-related distress, health-related quality of life, food cravings, and emotional eating.
- Weight: All participants will have their weight measured at their DXA appointment. Participants will be asked to weigh themselves at home and provide the results to the study team. Those who do not have access to a scale at home will be mailed one to use throughout the trial.

Approximately 1–2 weeks before the class start date, participants who have completed all baseline steps will be reminded of the dates and time of the study classes, and study staff will ask them to confirm their availability and interest. They will then be randomized to one of the two diet groups and informed of their assignment.

##### Medication consultations

The study includes steps to limit the risk of hypoglycemia if participants substantially reduce their glucose levels due to their assigned diet group. Participants who are using glucose-lowering medications that have significant risk of hypoglycemia, such as insulin or sulfonylureas, will attend a video call prior to the start of classes with the study doctors. They will be notified that study doctors may advise medication changes throughout the trial. Study physicians will review the signs and symptoms of low blood sugar and will provide recommendations for monitoring blood sugar throughout the trial, with an emphasis on the importance of monitoring as participants transition to their new diet. Participants will also be informed about how and when to contact the study team.

**Fig. 1:**
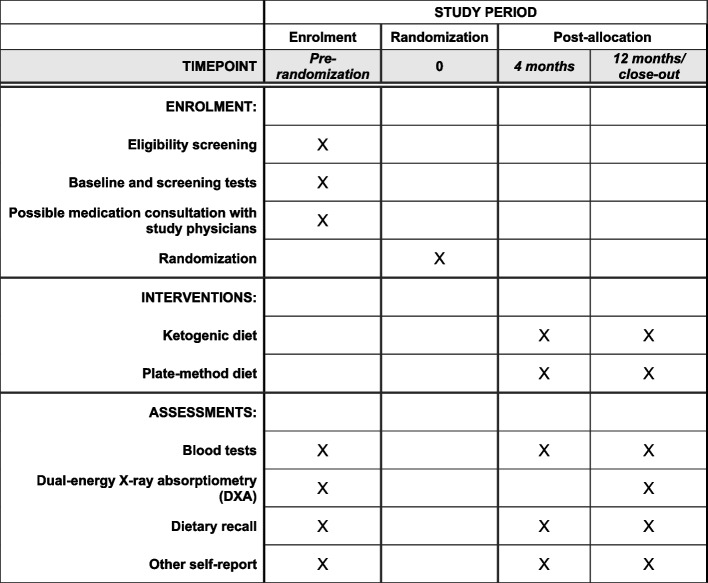
Schedule of enrolment, interventions, and assessments

Figure 1 shows the SPIRIT figure with the schedule of enrolment, interventions, and assessments. Table 2 shows when participants complete these and other measures in LEGEND.

**Table 2:** Summary of measures by aim and month

**Aim/domain**	**Measures**	**Baseline**	**4 months**	**12 months**
**Aim 1: Health effects**				
*Physical health*	Blood tests for HbA1c (primary outcome), inflammation (hsCRP), weight	x	x	x
*Medication-related changes*	Medication use questionnaire for diabetes medication amounts [21]	x	x	x
*Psychological effects*	Diabetes-related distress [27], health-related quality of life (PROMIS-29) [28], craving for sweet [31], and emotional eating [32]	x	x	x
**Aim 2: Potential adverse effects**				
*Lipids*	Nuclear Magnetic Resonance lipid profile (Labcorp LipoProfile)	x	x	x
*Bone density*	Bone turnover markers (s-PINP and s-CTX) and dual-energy X-ray absorptiometry (DXA)	x	x (no DXA)	x
*Diet-related symptoms*	Self-report	x	x	x
*Decreased vegetable consumption*	Fruit and vegetable consumption by 24-h diet recall.	x	x	x
**Aim 3: Assess possible moderators**				
*Hypothesized moderators*	Insulin resistance, body mass index, and sex	x	x	x
**Other outcomes**				
*Dietary adherence and changes*	Self-report and 24-h dietary recall	x	x	x
*Physical activity and sleep*	Self-report [44, 45]	x	x	x
*Intervention satisfaction*	Self-report		x	x

### Sample size {14}

When calculating power for the primary outcome, we base all our calculations and overall study enrollment on a target sample size of 180. Assuming a 20% attrition rate, we would retain 144 participants at 12 months. Although we will use mixed effect models and include all follow-up data available on the full number participants in primary analyses, for sample size calculations, we use somewhat simplified analysis approaches, assuming a *t*-test on change in continuous outcome measures and based on 80% retention. Our primary analysis approach is based on mixed effects models and will include data from all participants including those who have partial follow-up data which should result in comparable or increased statistical power. Change in HbA1c levels is our primary outcome measure. We will have 90% power to detect a statistically significant (*p* < 0.05) difference in the mean change in HbA1c levels between intervention groups of 0.25% or greater.

### Recruitment {15}

To reach the target sample size of 180, we plan to recruit over 3 years. We will use electronic health records to identify potentially eligible participants, who will be sent invitations to join the study either by email (e.g., MyChart emails) or through physical letters using the University of Michigan and University of California, San Francisco, health systems. We will also advertise on social media to reach community populations living in the Ann Arbor, Michigan, and San Francisco, California, areas. At both sites we will post flyers in health care and community sites.

## Assignment of interventions: allocation

### Sequence generation {16a}

Eligible participants will be randomized in a 1:1 ratio to one of two groups based on a computer-generated (Python v3.7) permuted block randomization sequence for well-balanced assignments that minimizes the ability to anticipate assignment. Randomization is stratified by baseline HbA1c level (6.5–7.9%; 8.0–11.9%), to ensure a balance of the two interventions within each stratum.

### Concealment mechanism {16b}

The allocation sequence is implemented via a Salesforce interface that conceals the sequence until the interventions are assigned to a participant.

### Implementation {16c}

The study statistician will generate the allocation sequence. The allocation sequence will be implemented via a database interface (Salesforce) that conceals the sequence until an intervention arm is assigned to a participant. Study staff will then inform participants of the intervention arm assignment.

## Assignment of interventions: masking

### Who will be unmasked {17a}

We will not mask participants to treatment arm before initiation of intervention. Study physicians will not be masked to diet group, as they will know which group a participant is in when they make medication adjustments at baseline or during the intervention phase. We will not mask study staff to intervention group. Data analysis will be performed by the study statistician, who will be masked for planned primary and secondary outcome analyses, then may be unmasked. Laboratory staff collecting clinical outcomes (i.e., blood tests at Labcorp, DXA scans, and 24-h dietary recalls) will be masked to group assignment. Participants will also complete self-report questionnaires online, and the completion of these questionnaires will be monitored by unmasked study staff.

### Procedure for unmasking if needed {17b}

We do not anticipate a need for unmasking the study statistician or above-mentioned individuals, particularly as participants are aware of the nutritional intervention they are receiving, and we have thus not planned a procedure for unmasking.

## Data collection and management

### Plans for assessment and collection of outcomes {18a}

Study staff will review all assessment data for accuracy and completion, and will monitor loss to follow-up and missing and incomplete data.

### Plans to promote participant retention and complete follow-up {18b}

Payments will be provided to participants to promote retention: $35 at baseline for completing the DXA, $40 at 4 months for completing all 4-month measurements, $50 at 12 months for completing the DXA, and $45 for completing all other measurements. Participants will be paid via Amazon gift card or a visa gift card. We will provide lab results to participants at each time point via secure email.

### Data management {19}

Trial data will be collected by trained research assistants and study coordinators using questionnaires (online via Qualtrics). Laboratory data (Labcorp) will be transferred via electronic files and integrated into the study database, as will results from DXA scans (UCSF or University of Michigan Radiology). Protocol deviations will be captured by regular review of cases during the enrollment process to ensure that eligibility criteria are met before randomization. Study data will be stored using a HIPAA-compliant database (implemented in Salesforce) that uses cloud-based storage. The data system allows for specified ranges and automatic calculations to reduce entry errors. Data will be cleaned by investigators upon completion of data collection to ensure high quality.

### Confidentiality {27}

All surveys and forms will be deidentified and coded with a unique participant number.

### Plans for collection, laboratory evaluation, and storage of biological specimens for genetic or molecular analysis in this trial/future use {33}

Participants will have blood samples drawn at baseline and 4 and 12 months later. Blood samples are drawn, analyzed, and then destroyed by Labcorp.

## Statistical methods

### Statistical methods for primary and secondary outcomes {20a}

Outcome analyses will be performed by a dedicated biostatistician. An intention-to-treat analysis will be performed on all randomized patients and a per-protocol analysis will be performed on patients who adhered to their assigned diet. We define dietary adherence as reporting an average score of 7 or higher (scale of 0–10) for the level of dietary adherence on the final four surveys for which we asked about dietary adherence.

Descriptive statistics will be calculated for all outcome measures at each time point. Continuous variables will be reported using means, standard deviations, medians, and interquartile ranges, depending on the distribution. Categorical variables will be described using frequencies and percentages.

Our primary outcome is to compare HbA1c levels in participants in the two diet groups. Our primary analyses for HbA1c and other quantitative outcome measures will use random-intercept-random-slope mixed effects models to estimate differences between study arms in mean change in outcomes at 4 and 12 months. We will include several pre-specified covariates that we will adjust for to address any potential imbalances in randomization: sex, age, insulin use at baseline (yes/no), and education (as a marker of socio-economic status). When assessing outcomes such as HbA1c or weight over time, linear models that assume a constant association between time and outcome are unlikely to be appropriate. We anticipate much of the benefit to occur in the initial intervention period, with lesser benefit or even loss of benefit over time. To address this, we will use a linear spline of time, which uses knots to allow changes in slopes at key points, in this case, the end of the main intervention period. The overall issue of whether a very low-carbohydrate diet influences HbA1c primarily through weight loss or through other mechanisms is not the primary focus of this trial: both pathways are likely important, but the clinical result in glycemic control at the 12-month timepoint is the main focus. To explore the potential associations between weight loss and HbA1c; however, we will assess changes in HbA1c before and after statistically accounting for change in weight to help identify whether diet intervention group is affecting glycemic control via weight loss or via other mechanisms.

We will use similar analysis strategies for other continuous outcomes, using linear mixed-effects models with adjustments for the covariates noted for the primary outcome. As weight loss is a well-known predictor of bone loss, and one of the diet groups may lose more weight, on average, than the other, we will statistically account for weight loss in analyses of bone mineral density and bone turnover markers to determine if diet group predicts changes in these measures independent of weight loss.

We will summarize symptom checklists by presenting the number and percent of participants who report each symptom more than 1 day in the previous month at baseline, 4 months, and 12 months. We will visualize data using stacked bar graphs that allow for visual comparison of presence and frequency of individual symptoms across intervention arms. We will use chi-squared tests to compare the percent of participants achieving diabetes remission or reversal.

### Sensitivity analyses

A set of sensitivity analyses will be conducted with models adjusting for baseline outcome (using an ANCOVA approach) and pre-specified baseline covariates as described above (sex, age, insulin use at baseline, and education), to evaluate whether baseline differences impact estimates of treatment effects. Separate models will be run for each outcome time (4 month and 12 month), with baseline outcome measure included as a predictor, along with time, diet group, and their interaction, and covariates as described. A random intercept will be included to account for clustering by intervention group. Estimates of change within diet group, and differences between diet groups in change, will be derived from these models.

### Interim analyses {21b}

This trial has no planned interim analyses or stopping rules. Although our outcome, HbA1c, indicates long-term risk of clinical events due to diabetes, it is not an outcome that would justify early stopping rules such a trial. The data and safety monitoring board will review any intervention-related serious adverse events, to determine whether the study should be stopped.

### Methods for additional analyses (e.g., subgroup analyses) {20b}

We will explore whether baseline characteristics modify the benefits of diet group assignment on HbA1c by assessing whether there appear to be differences in the magnitude of HbA1c changes between diet intervention groups across three pre-defined sub-groups (levels of insulin resistance, levels of obesity, and women versus men). Linear mixed models similar to those describe above will be used, with the addition of candidate moderators (each in separate models), and interactions between the moderator, intervention arm, and time. These models will be used to estimate change in the outcome within subgroups and differences in change between subgroups. The focus of these exploratory analyses will be the magnitude and direction of change within and between subgroups. We will also report the statistical test on the interaction term, which represents an overall test of the moderation effect.

### Methods in analysis to handle protocol non-adherence and any statistical methods to handle missing data {20c}

We will examine patterns of missing data and proportions and compare baseline characteristics of participants with and without missing data to evaluate potential impact on estimates. In addition, our primary analyses for outcomes will use random-intercept-random-slope mixed effects models, to estimate differences between study arms in mean change in outcomes at 12 (primary) and 4 (secondary) months. Mixed effects models using maximum likelihood estimation are relatively robust to the effects of missing data, and they allow appropriate assessment of repeated measures.

### Plans to give access to the full protocol, participant level-data {31c}

Upon publication of the trial’s pre-specified outcomes, a de-identified dataset will be provided to other investigators upon reasonable request with the agreement of the trial steering committee, providing the request is in alignment with institutional review board protocols.

## Oversight and monitoring

### Composition of the coordinating center and trial steering committee {5d}

This is a two-center trial. The primary decision-making body of this study is the investigative team comprising the principal investigator (PI; Saslow), University of California PI (Hecht), and the co-investigators. The PI and site PI are responsible for the overall management of the study. They coordinate the operations of the study, review issues that arise in the conduct of the study in between investigative team deliberations, and bring issues to the investigative team for decision. The PI (Saslow) serves as the liaison with the funding body, including submission of annual reports and providing overall management of the fiscal and administrative operations, and is also responsible for the study coordination and implementation at the University of Michigan site. The site PI (Hecht) is responsible for the study coordination and implementation at the University of California site.

The trial steering committee will consist of the principal investigator (LS), the UCSF site principal investigator and trial co-investigator (FH), trial coordinator (PM), and study statistician (WH). They will meet weekly with study staff to discuss study implementation and adverse events and monthly with other study investigators and staff to discuss overall study issues and evaluate the progress of the trial. There will be ongoing communication by way of group email for needs such as enrollment questions and addressing issues such as suggesting medication changes for participants.

The project coordinators and research assistants are responsible for the day-to-day operations of the study, including recruitment, data collection processes, and intervention process. They also coordinate institutional review board revisions and data monitoring reports and document completion of the trainings. Staff are responsible for recruiting and screening the participants, obtaining informed consent with participants, and scheduling and conducting follow-up assessments. Interventionists are responsible for the treatment implementation. The lead project manager supervises the development of the study data tracking system and surveys.

### Composition of the data monitoring committee, its role, and reporting structure {21a}

We have created a 3-person data safety monitoring board. We will hold board meetings twice a year via videoconference to review the reports of recruitment, retention, and safety information.

### Adverse event reporting and harms {22}

In the case of a serious adverse event that is likely to be related to study participation, we will call for a special closed meeting of the data and safety monitoring board to review any needed changes or early stopping of the trial. Here, an adverse event includes any event that causes or increases the risk of harm to the participant or others. Serious adverse events include any events that result in death, inpatient hospitalization or prolongation of existing hospitalization, a persistent or significant disability or incapacity, or a congenital anomaly or birth defect. The study team reviews all potential adverse events reported by study participants and determines their relatedness to diet or study intervention, expectedness, and severity.

Either dietary approach in the trial could lower glucose levels, which could increase the risk of hypoglycemia among individuals using glucose-lowering medications, such as sulfonylureas or insulin, unless appropriate medication adjustments are made. Therefore, one internal medicine physician (FH) and one endocrinologist (SK) with experience in medication management in people with type 2 diabetes who have transitioned to reduced-carbohydrate diets will serve as study physicians. Initial medication consultations will be done via video conference with a study physician and study staff member prior to the start of classes. The participants who will receive medication consultations include any participant taking glucose-lowering medications such as insulin or sulfonylureas that may put the participant at risk of hypoglycemia regardless of which diet intervention they are assigned to. During 15-min consultations, participants are informed about the possible medication adjustments that might be made, the possible symptoms of hypoglycemia, and to contact the study team with questions about symptoms or medications. These participants also receive individually tailored plans to check their blood glucose levels, typically at least daily.

During the initial roughly 3 months of the intervention, before each week’s session, participants will receive a brief online survey about their blood glucose levels during the past week. This includes questions about whether they had any values below 80 mg/dL, between 80 and 110 mg/dL, and/or above 180 mg/dL. Their reported blood glucose levels will be reviewed by a study staff member as well as by the study physicians to determine whether any medication changes are needed. If a plan for recommending a medication change is agreed upon, a study staff member will relay the recommendation to the participant. Significant symptoms or other health issues reported by participants, whether related to the study intervention or not, will be recorded in the trial database and followed up by the study team as needed.

We will provide participants’ PCPs with information regarding the study once the participant has initiated the process for enrollment. If we recommend medication reductions, we will ask the participant to inform their PCP, and we also notify the PCP if the physician requests that we provide updates to them throughout their patient’s experience in the trial.

### Frequency and plans for auditing trial conduct {23}

Study investigators and the data and safety monitoring board will closely monitor the trial, meeting twice a year. The study team will provide annual progress reports to the institutional review board and to National Institute of Diabetes and Digestive and Kidney Diseases (NIDDK), the study sponsor.

### Plans for communicating important protocol amendments to relevant parties (e.g., trial participants, ethical committees) {25}

Any trial design changes, such as for trial eligibility, will be reviewed by the institutional review board. If the changes are approved by the institutional review board, they will be updated in ClinicalTrials.gov.

### Dissemination plans {31a}

The results of the trial will be presented at conferences, uploaded to ClinicalTrials.gov, and published in peer-reviewed publications. All final peer-reviewed manuscripts that arise from this proposal will be submitted to the digital archive PubMed Central. Wherever applicable, data will be deposited to appropriate public repositories.

## Discussion

This paper describes the protocol of the LEGEND trial, a randomized, controlled trial comparing two dietary approaches with varying carbohydrate intake for the nutritional management of type 2 diabetes. These two approaches include a very low-carbohydrate diet and a moderate-carbohydrate plate-method diet. The trial is aimed at addressing important knowledge gaps that have been noted by the American Diabetes Association and other experts in making clinical guidelines for the nutritional management of type 2 diabetes in regard to macronutrient content. We aim to address important knowledge gaps by conducting trial with a 12-month follow-up to assess effects of two dietary approaches with varying carbohydrate intake on clinical in type 2 diabetes; this trial will have adequate statistical power to detect clinically meaningful differences in HbA1c levels. We have included support for ongoing adherence to the two dietary approaches to reduce the chance that declines in intervention benefit are due to limited support for long-term adherence. We also include measures to assess potential adverse effects of the very low-carbohydrate nutritional plan that have not been carefully assessed, to our knowledge, in people with type 2 diabetes, such as effects on bone mineral density and bone turnover markers.

There are several limitations inherent in this trial. We are not able to mask study participants or study physicians to diet group allocation. We aim to partially limit any effects of lack of masking by having laboratory staff, who are masked to the diet allocation group, perform assessments (i.e., 24-h diet recalls, blood draws, DXA scans) and performing initial study analysis with the study statistician masked to diet group allocation. We aim to address additional biases as much as practically possible by randomizing and concealing of allocation where possible, employing strategies to minimize and manage incomplete outcome data, having appropriate duration of follow-up, and making a priori specifications of all primary and secondary outcomes as detailed in this study protocol and in our clinical trial registration.

To our knowledge, the LEGEND trial is the first, randomized controlled trial of this size to compare the effects of a very low-carbohydrate diet to a moderate-carbohydrate plate-method diet with a 12 month of follow-up. This trial research addresses important gaps in the evidence base for the nutritional management of type 2 diabetes and holds the potential to strengthen evidence-based approaches to improve type 2 diabetes outcomes.

## Trial status

Protocol version 1.3; December 8, 2022. Recruitment was initiated in March 2022, and the approximate date for completion is July 2025.

## Data Availability

The final trial data set required to support the protocol can be provided upon request.

## Abbreviations

BMI: Body mass index
DXA: Dual-energy X-ray absorptiometry
HbA1c: Glycosylated hemoglobin
HDL: High-density lipoprotein
hsCRP: High-sensitivity C-reactive protein
LEGEND: Lifestyle Education about Nutrition for Diabetes
LDL: Low-density lipoprotein
NIH: National Institutes of Health
